# The effect of lateral thrust on the progressive slope failure under excavation and rainfall conditions

**DOI:** 10.1007/s11069-024-06635-9

**Published:** 2024-05-02

**Authors:** Xiang Yu, Tao Zhao, Bin Gong, Yongjun Zhang, Chun’an Tang, Yu Luo

**Affiliations:** 1 State Key Laboratory of Coastal and Offshore Engineering, Dalian University of Technology, Dalian, 116024 China; 2 Department of Civil and Environmental Engineering, Brunel University London, London, UB8 3PH UK; 3 School of Civil Engineering, Qingdao University of Technology, Qingdao, 266520 China; 4 Institute of Mountain Hazards and Environment, Chinese Academy of Sciences, Chengdu, 610041 China

**Keywords:** Slope failure, Lateral thrust, Multiple failures, Excavation, Rainfall

## Abstract

Large landslides can involve the multiple failures of regional slopes. To understand the effect of lateral thrust caused by the failure of one slope on its surroundings, the failures of two adjacent highway slopes in Guangdong Province, China, were investigated in detail. The interactive failure processes and landslide morphological characteristics of the two slopes were first analyzed based on the on-site investigation. Then, a plane mechanical model of a large-scale slope was established to evaluate the significant influence of the lateral thrust generated by the west slope acting on the east excavated slope. Furthermore, the extrusion effect of the west slope was modelled under the alternate excavation disturbance and rainfall by transferring the thrust forces onto the interface elements, and the induced failure mechanism and instability mode of the east slope under lateral thrust were reproduced numerically. The results show that the compression-shear failure occurred at the middle and rear slope bodies because of the lateral thrust, which led to the formation of a thrust landslide and the final instability of the east slope.

## Introduction

Large-scale landslides with multiple slope failures generally involve large area, wide range, complex terrain, multi-level sliding surfaces and directions. It is always difficult to determine the depth and range of sliding masses, causing great challenges in slope designing, monitoring, landslide early warning and mitigation. These large-scale slopes over the height of 100 m have been encountered in mountainous regions (Xie et al. 2020; Tao et al. 2021; Tu et al. 2020). Because of the great gravitational potential energy, the large-scale landslides can cause massive economic losses and casualties (Zhang et al. 2017; Cui et al. 2018; Ji et al. 2020; Guo et al. 2020). In recent years, the research on large-scale landslide can be divided into two categories. The first is to predict the future deformation and assess the landslide risk of a slope with potential instability possibility based on geological structure, displacement and landform. According to different technical means, this category can be divided into: (1) geological structure analysis, i.e., the landslide direction and distance are predicted by the strike and length of the fault zone (Qi et al. 2021; Zhao et al. 2019; Liang et al. 2022); (2) remote sensing surveying and mapping, i.e., the slope surface deformation and landform at different stages are compared to determine whether there is landslide risk, and to estimate the possible damage area (Liu et al. 2021a, b; Ma et al. 2021; Burrows et al. 2019; Takada and Motono 2020; Guo et al. 2021); (3) artificial intelligence (AI), i.e., according to displacement, stress and other data, the emerging AI technologies are used to established the related model to predict the slope stability, such as machine learning and artificial neural network (Liang et al. 2014; Gong 2021; Palau et al. 2022); (4) weight analysis, i.e., the evaluation model considering factors such as slope height and gradient is built to qualitatively assess the risk level of slope failure (Román 2021; Xu et al. 2021; Zhang et al. 2020; Gu et al. 2021). Generally, the failure mechanism cannot be directly included in the first category of research, and its main purpose is to avoid or reduce the impact of landslide. The second is aimed on clarifying the mechanical mechanism of slope failure. Some researchers have inversed the movement process of landslides and analyzed the dynamic mechanism of landslides according to the observed information, such as movement speed and accumulation thickness (Gong and Tang 2017; Gong et al. 2018; Huang et al. 2022; Zhuang et al. 2022; Gong et al. 2024). Meanwhile, the critical advances have been achieved in terms of describing the deformation and failure characteristics by field investigation and analyzing the causes and mechanical behaviors by the indoor test of sliding-zone soil (Li et al. 2021; Wang et al. 2022a, 2022b; Feng et al. 2022; Chen et al. 2022). However, the trigger mechanisms of large landslides that involve the multiple failures of regional slopes under complicated anthropogenic and environmental factors remain unclear.

During progressive landslide, the local soil mass is generally first destroyed, and a plastic zone may form. Then, the rest parts of the slope are pulled or pushed to make the shear zone expand, finally leading to slope instability (Darban et al. 2019; Bastian et al. 2021; Du et al 2021). Before a large landslide, there are often precursors such as accelerated deformation (Xing et al. 2021), terrain and landform change (Ortuño et al. 2017), crack propagation (Luo et al. 2019), and micro-seismic event gathering (Xie et al. 2022), which indicate that a large landslide has a specific breeding and evolution process. In terms of space, since the soils at the sliding surface will experience elastic stress, elastic–plastic stress, critical stress and post-failure stress, the slope can also be divided into the stable zone, unstable zone, critical zone and unstable zone (Lu et al. 2021). In terms of time, with the reduction of the factor of safety, the slope will enter the creep, extrusion, sliding and severe sliding deformation stages successively (Wang et al. 2017). The expansion of unstable zone and the change of slope deformation require the continuous action of a single trigger factor or the coupling action of multiple trigger factors. Especially, excavation and rainfall represent the two main causes of slope failure worldwide (López et al. 2020; Yu et al. 2021; Troncone et al. 2022; Zhao and Zhang 2022). The former can cause the destruction of foundation pit, mine and cutting slope, while the latter can cause the instability of reservoir bank slope (Chandel et al. 2021; Su et al. 2022; Liu et al. 2021a, b; Zhang et al. 2022) and dam (Hu et al. 2021; Su et al. 2021). Then, it is of great practical significance to carry out the mechanism research of large landslides that involve the multiple failures of regional slopes under excavation and rainfall.

As an inevitable key process in slope engineering, the impact of excavation mainly contains two aspects: (1) the excavation unloading can reduce the stress level and redistributes the stress distribution (Fang et al. 2022; Yang et al. 2023). As the in-situ horizontal stress of the soil is significantly reduced, and the gravity stress does not change, the resulting shear stress can exceed the peak strength, leading to shear failure (Kawadas et al. 2020; Yu et al. 2021); (2) the excavation exposes the stratum and forms a free face, which causes the slope to lose effective mechanical support and the toe to be damaged due to stress concentration (Stark et al. 2005; Robert 2017; Li et al. 2020). The damaged toe gradually pulls the upper soil mass and finally causes the slope instability (Yang et al. 2022; Zhang et al. 2018). In addition, excavation disturbance will cause rebound effect, leading to rapid deterioration of rock and soil mass quality. Zhu and Huang (2019) found that the cohesive force of the soil sample under unloading condition was significantly reduced. The redistribution of slope stress field caused by excavation will make the soil show the strain softening characteristics and promote the development of landslide (Sabatini and Finno 1996).

Compared with the human engineering activities, the impact of rainfall on slopes has been observed for a longer history. According to the statistics of ancient landslides worldwide (Pánek 2019), the landslides in the late Pleistocene ice age were dormant. After entering the Holocene, with the climate becoming warm and humid, the landslides in the mountains, tropical and coastal areas were revived and activated. From the microscopic perspective, the pore network between rock and soil particles serves as a pathway for rainfall infiltration and flow. When rainfall infiltrates rock and soil, it creates both high saturation areas with high hydraulic pressure and low saturation areas with low hydraulic pressure. Under pressure difference, rainfall flows from high saturation areas to low saturation areas through pore channels until the pressure on both sides is equal (Song et al. 2024; Kong et al. 2021, 2022). From the macroscopic perspective, there are generally two ways of rainfall infiltration into slope: (1) for exposed artificial or natural slopes filled by geo-materials with low permeability coefficient, rainfall can infiltrate into the shallow area near slope top and surface, and the middle and lower parts are often not saturated (Tang et al. 2020; Paronuzzi and Bolla 2023; Dolojan et al. 2023; Tozato et al. 2022); (2) for slopes filled by geo-materials with high permeability coefficient and shallow buried groundwater, atmospheric precipitation can supplement groundwater, causing saturation in the middle and lower parts of a slope, while the upper part is in an unsaturated state (Song and Tan 2020; Tang et al. 2015).

The influence of the two rainfall infiltration modes on slope has similarities and differences. The main similarities include: (1) rainfall can reduce the matrix suction of unsaturated soil and produce a transient saturated zone, softening the rock and soil mass (Fredlund and Lim 1994; Oh and Lu 2015); (2) infiltrated rainfall can increase the unit weight of geo-materials, make the sliding moment greater than the anti-sliding moment, and cause slope instability (Qiu et al. 2022; He et al. 2022); (3) the rainfall can increase the pore water pressure and reduce the effective stress, thus weakening the shear strength of rock and soil mass (Bishop 1959). The main differences include: (1) for the shallow infiltration type, the rainfall on the slope surface will erode the toe, causing the loss of effective lower support for stabilizing the slope. The rainfall near the slope top will gather inner the tension cracks, forming hydrostatic pressure and squeezing the rock and soil mass (Yang et al. 2019; Shi et al. 2012); (2) for the water-level supplement type, the formation or rise of groundwater level will reduce the friction resistance of the middle rock and soil mass along the bedrock surface (Yang et al. 2018; Li and Ju 2016), causing deep landslides. Besides, the seepage force generated by rainfall infiltration can cause soil piping (Wang et al. 2021; Saada et al. 2012), and the dry–wet cycle effect by rainfall and evaporation will cause the deterioration damage of geo-materials and reduce the long-term stability of slope (Xu et al. 2022).

Furthermore, the failure mechanism of a slope under the alternate excavation and rainfall is worthy of attention. In slope engineering, the excavation disturbance will result in the formation of bare slope, and the unloading-induced rebound effect will cause the initiation and propagation of cracks inside the slope, providing many effective infiltration channels for rainfall (Li et al. 2020; Shi et al. 2021). Hence, the subsequent infiltrated rainfall will further promote the development of cracks, drive the sliding surface to expand, and quickly transform the deformation localization into landslide (Yan et al. 2019). Under the action of the excavation + rainfall disaster chain, large slopes often slide in the form of multi-level and multi-region failures (Fan et al. 2018; Deng et al. 2021). After the instability of a single regional slope, the failed slope will experience lateral expansion and shear dislocation with its adjacent slopes. The generated extra force may induce further sliding and failure of its adjacent regional slope. This has been confirmed by the related geological structure analysis (Omeru et al. 2015), remote sensing mapping (Aslan et al. 2021; Qu et al. 2021; Zhang et al. 2016; Jiang et al. 2022), multi-temporal landslide inventory analysis (Lin et al. 2022), deformation monitoring and laboratory tests (Schulz et al. 2018). However, the influence mechanism of lateral thrust generated by regional slope failure on adjacent slopes is still unclear.

In this study, the large slope engineering affected by the excavation + rainfall disaster chain in Guangdong, China as was investigated comprehensively. Through the geologic survey and on-site investigation, the squeezing effect of the regional slope failure on the adjacent slope was analyzed, and the interactive failure processes and landslide morphological characteristics of the two sub-slopes were discussed. Then, after the back calculation of the saturated shear strength parameters, the extrusion effect of the west slope was simulated numerically under the alternate excavation disturbance and rainfall by transferring the thrust forces onto the interface elements. Finally, the failure mechanism and instability mode of the east slope under lateral thrust were studied.

## Overview of the highway slope

### Basic characteristics

**Fig. 1:**
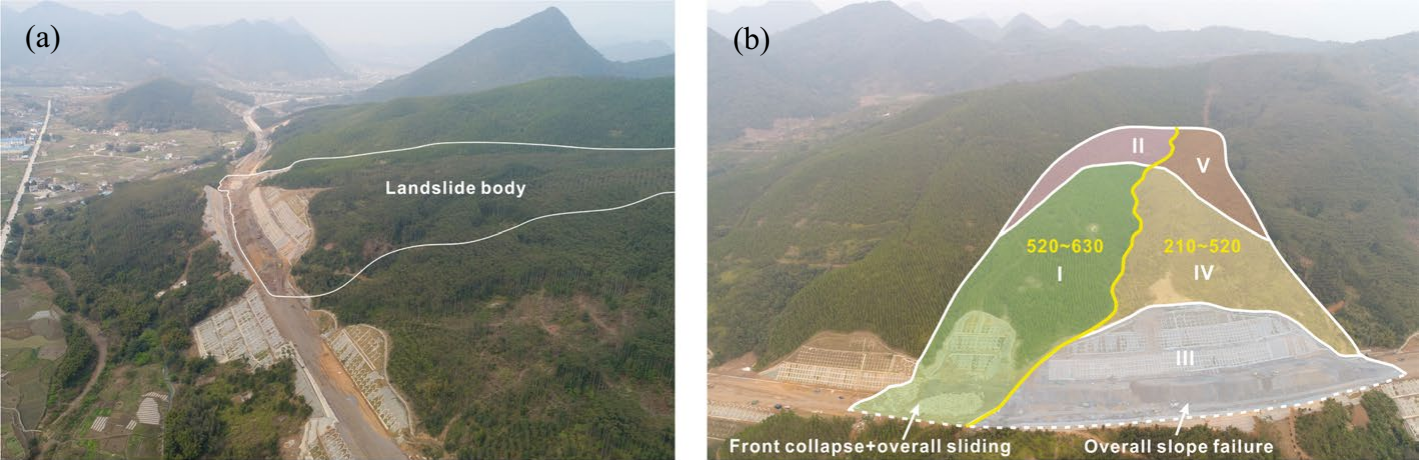
Remote sensing images of the K112 + 210–K112 + 630 highway slope: **a** side view and **b** front view and geological division

The K112 + 210–K112 + 630 large-scale slope of the highway construction project in Guangdong Province, China, has a natural slope angle of 17°–23°. The slope is high in the north and low in the south, as shown in Fig. 1a. The elevation of the northern mountain top is about 355 m, and the lowest elevation of the southern highway is 270 m. The slope area is located at the southeastern edge of the South China Plate, and generally presents syncline structures, as shown in Fig. 1b. Due to the intense tectonic movement, the occurrence of rock formations has undergone significant change, with observed local wrinkling. According to the measurement at the bedrock outcrop, the attitudes of the rock strata in the southern and northern part are 205–250°∠15–53° and 200–230°∠19–61°, respectively, which are unfavorable for slope stability.

The slope is divided into two sub-slopes by a large gully develops in the middle as illustrated by the yellow line in Fig. 1b. The gully first extends from the rear to the shoulder of the slope, and then turns westward to the slope toe, dividing the large slope into two sub-slopes. The plane shape of the west K158 + 520–630 slope is pentagon, with an inclination of 213°. The front excavated toe is 40 m high with the slope ratio of 1:1–1:1.25. The east K158 + 210–520 slope is boot-shaped with the slope inclination of 180°. The maximum height of the excavated toe is 31 m, with the slope ratio of 1:1–1:1.5.

### Landslide process and characteristics

**Fig. 2:**
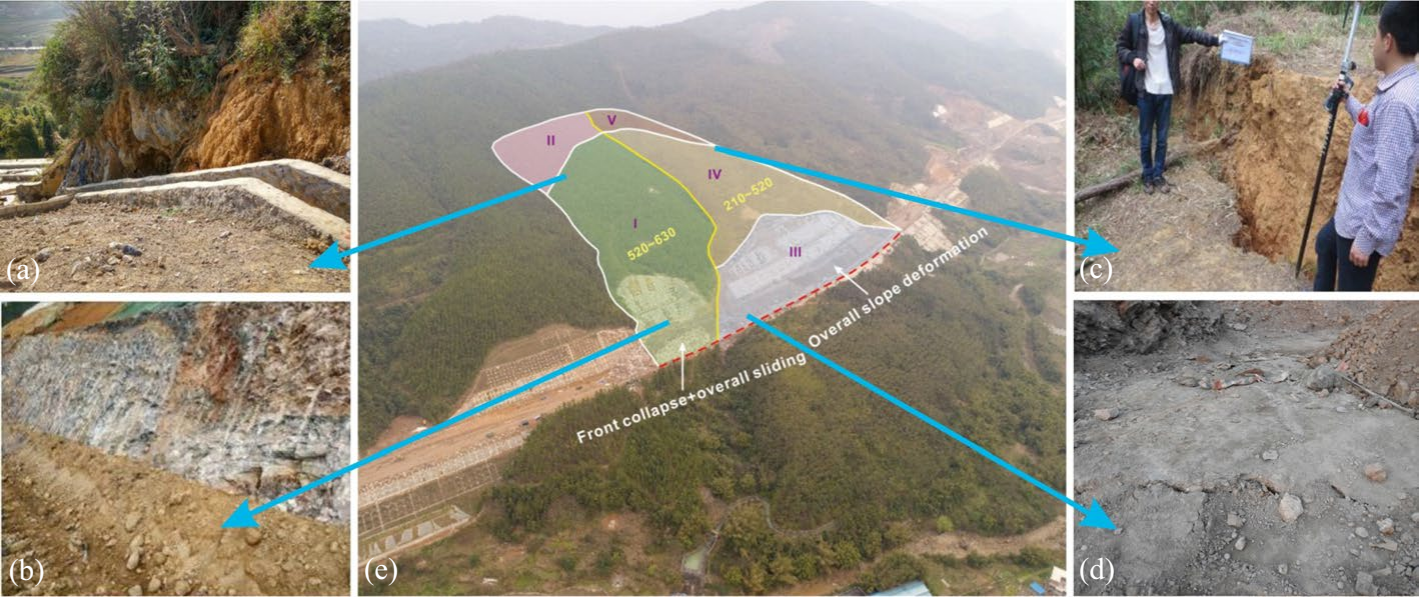
On-site investigation of the landslide area: **a** landslide bed, **b** shear outlet, **c** steep scarp and **d** uplift of road surface

The construction of the west and east slopes began in December 2015, and the excavation and slope protection were completed in April and May 2016, respectively. Several heavy rainfalls occurred in the slope area from July to October 2016. On December 11, 2016 the local failure of the west slope happened. The rear masses moved downward for 2 m, causing the exposure of the landslide bed, as shown in Fig. 2a. Then, the excavated slope collapsed, the mortar rubbles were wrapped in the sliding masses and covered the road and lower mountains, and the shear outlet appeared at the slope toe, as shown in Fig. 2b. On December 13, 2016, the east slope largely deformed, a large amount of through cracks appeared 150–220 m behind the slope shoulder, with a width of 10–100 cm. Meanwhile, the soil near the landslide boundary was clearly staggered, forming a steep scarp with a maximum height of about 1.1 m, as shown in Fig. 2c. Later, the front sliding body moved downwards and buried nearly half of the road. The road near the landslide has a 40 cm uplift, as shown in Fig. 2d.

According to the on-site investigation, the sliding direction of the two sub-slopes were consistent with the inclinations. Based on the slope deformation, the whole large slope can be divided into I–V zones, as shown in Fig. 2e. Zone I was the main sliding area of the west slope, whose front and rear boundaries were the shear outlets at the slope toe and the landslide cliff which were 180–220 m behind the slope shoulder. Zone II was the traction deformation area, and the rock and soil mass in this zone partially deformed. Zone III was a jointly affected zone. Clearly, the west “boot head” and the rest were affected by the sliding deformation of Zones I and IV, respectively. The area was mainly composed of the east excavated slope. Zone IV is the main sliding area of the east slope, including the masses from the slope shoulder to the rear tension cracks and scarps. Zone V was the rear edge area of the east slope, and no obvious deformation was observed.

### Geological survey and analysis

**Fig. 3:**
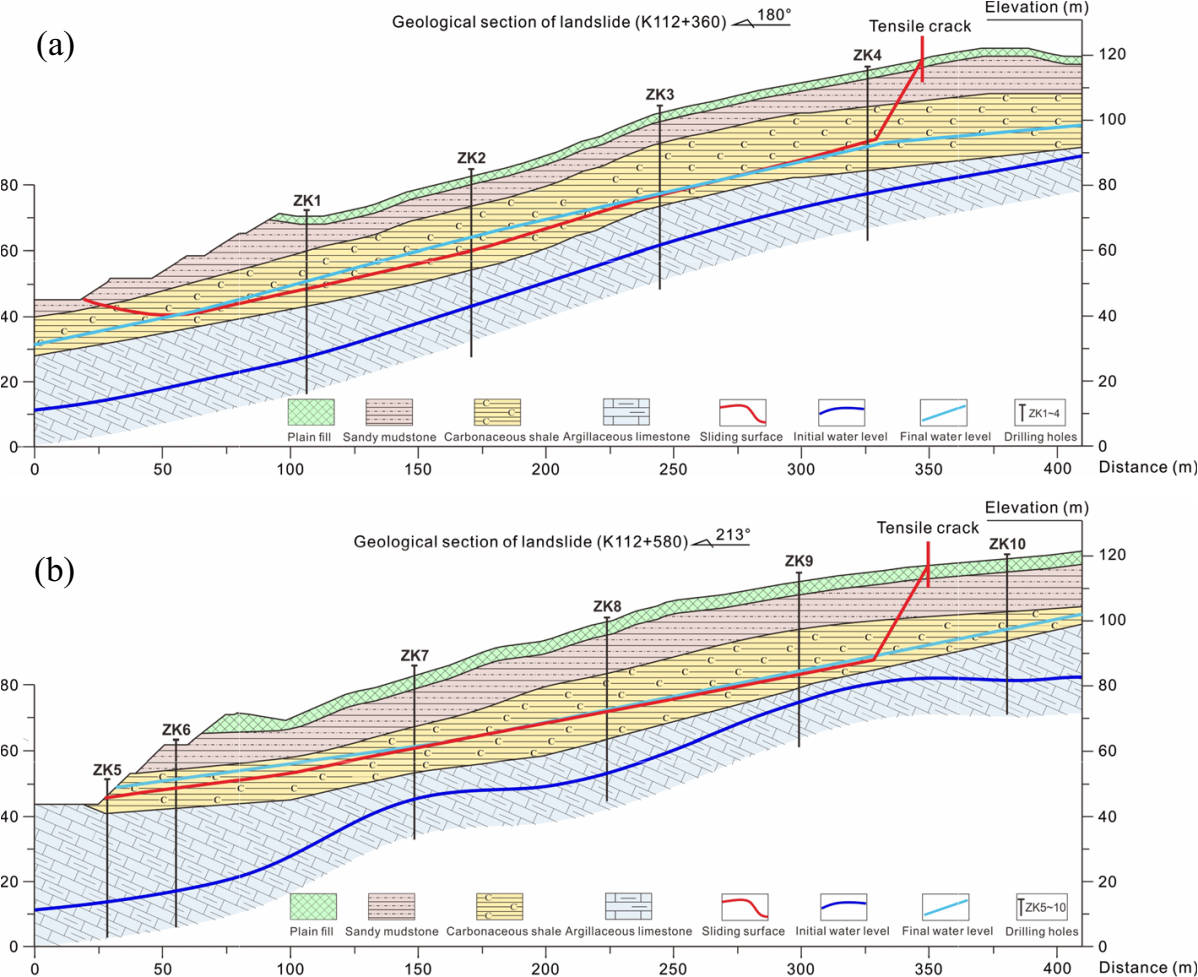
The typical geological sections: **a** K112 + 360 and **b** K112 + 580

In order to study the inducing factors of two landslides, the engineering geological mapping, geophysical prospecting and drilling exploration methods were used to comprehensively investigate the sliding surface, morphological characteristics, stratum distribution and hydrogeological condition. A total of 10 supplementary geological boreholes and 14 boreholes for groundwater level observation were implemented along the main slide lines of two slopes. Two typical geological sections (K112 + 360 and K112 + 580) from west to east are shown in Fig. 3a and b.

**Fig. 4:**
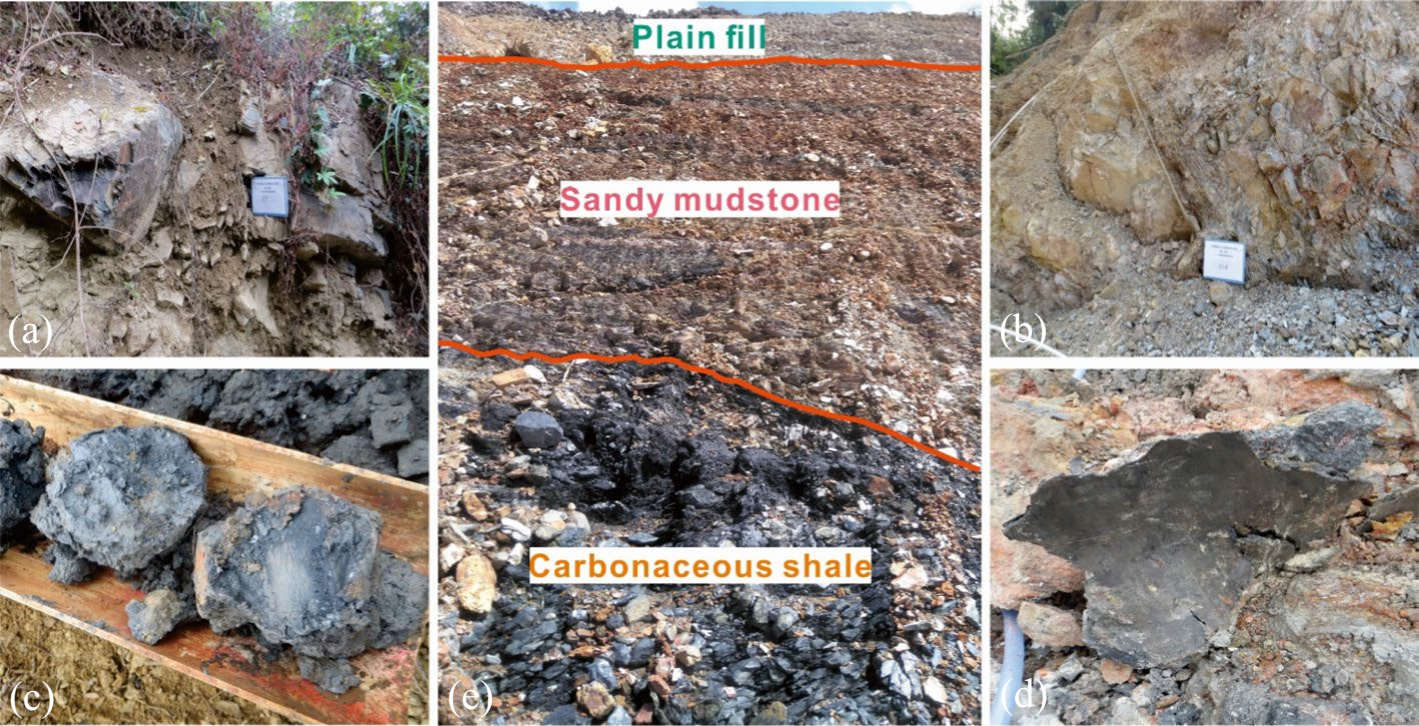
The geological survey of two regional slopes: **a**, **b** the exposed sandy mudstones, **c**, **d** the drilling cores of carbonaceous shales, and **e** the exposed strata at the surface of the west excavated slope

The drilling exploration show that the strata is layered and inclined from southeast to northwest. The two landslide masses are composed of plain fills, sandy mudstones and carbonaceous shales from top to bottom, and both landslide beds are argillaceous limestones, as shown in Fig. 4. Based on the exposed bedrocks and drilling cores, the plain fills and argillaceous sandstones are dry and broken, as shown in Fig. 4a and b. In contrast, the carbonaceous shales are earthy and wet. The samples of the west slope show obvious slip marks, as shown in Fig. 4c. While the east ones show the wrinkle marks caused by compression, as shown in Fig. 4d. Furthermore, the sliding surfaces of two slopes are basically located in the middle shale layer, which are substantially consistent with the groundwater level rise from the argillaceous limestone layer, showing the characteristics of bedding sliding, as also indicated by the red lines in Fig. 3a and b.

Although both the east and west sub-slopes are subjected to bedding sliding, the diverse deformation characteristics of the sliding zone soils demonstrate that the failure modes and inducing mechanisms of the two landslides are different. For the west slope, based on the sliding traces of shale drilling cores and the external inducing factors of excavation and rainfall, it can be concluded that the failure type is traction landslide. Since the excavation slope rate is steeper than the natural slope rate, the engineering activity creates a void surface at the slope front, which makes the slope lose the adequate mechanical support and provide many infiltration channels for rainfall. Rainfall replenishes the groundwater level, causing the rise of the groundwater to the carbonaceous shale layer. This process reduce the shear strength and effective stress of the soil and leads to a weak zone. The front sliding mass gradually pulls the rear soils, which eventually results in the bedding sliding.

In terms of the east slope, from the wrinkle marks of the shale drilling cores, it can be seen that the soil mass at the slope front is crushed and damaged by the push of the rear blocks, showing the typical characteristics of thrust-type landslide. According to the field investigation, it is basically impossible for rainfall to infiltrate along the dense vegetations at the slope top and form the hydrostatic pressure to push slope bodies. Considering the successive failures of the east and west sub-slopes, as well as the special boot-shaped plane of the east slope, the sliding deformation of the east slope may be caused by the squeeze force of the west slope. Namely, the west slope was destroyed first and the generated volume thrust at the central boundary resulted in the large deformation and final failure of the east slope.

## Analysis method for the lateral thrust effect

### Influence of the lateral thrust

**Fig. 5:**
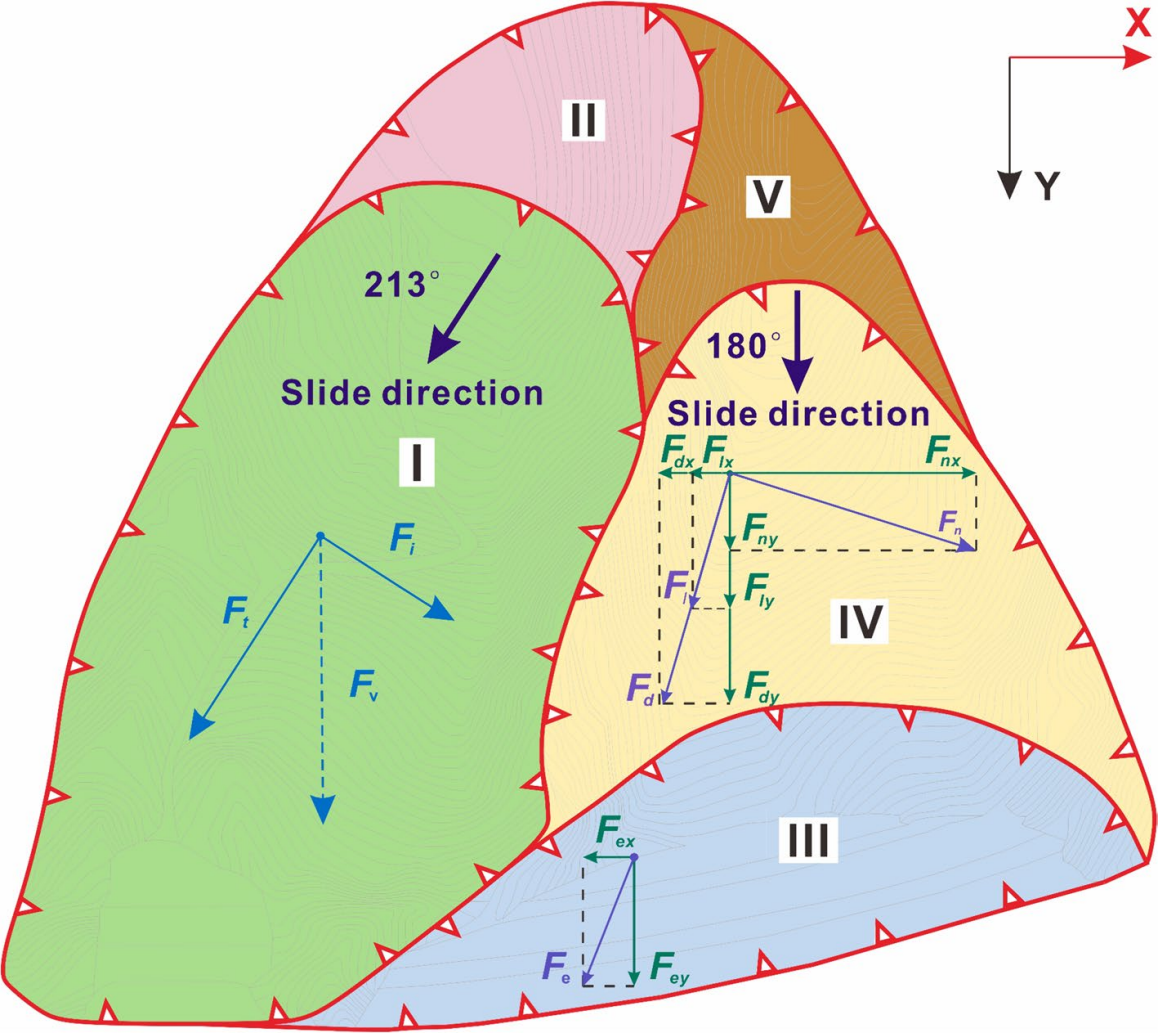
Schematic diagram of the lateral thrust of local landslides

According to the slope zoning, a plane mechanical model was established to study the influence of the west slope failure on the east slope, as shown in Fig. 5. Since Zones II and V were located at the trailing edge without large sliding deformation, they were not taken into calculation.

Figure 5 shows the slide directions. Along the west slope landslide direction, sliding masses generate a thrust *F*_*t*_. Meanwhile, the masses extrude the sides during the movement and produce an interface force *F*_*i*_ perpendicular to the thrust. Based on the site investigation, *F*_*t*_ > *F*_*i*_. Therefore, the direction of the volume force *F*_*v*_, combined by these two forces, should be skewed towards the direction of *F*_*t*_ and point towards the east excavated slope.

For the Zone III, because of the 'boot head' block part of the west slope, it undertakes the extrusion force *F*_*e*_ by thrust *F*_*t*_ without being affected by *F*_*i*_. To analyze the effect of lateral thrust on the east slope, the coordinate system is established by regarding the horizontal right direction as the positive *x*-axis direction and the slide direction of the east slope as the positive *y*-axis direction. The *F*_*e*_ is decomposed as *F*_*ex*_ and *F*_*ey*_, which can be expressed as:

1 $$\left\{ {\begin{array}{*{20}l} {F_{ex} = F_{e} \sin 33^{ \circ } \approx 0.54F_{e} } \hfill \\ {F_{ey} = F_{e} \cos 33^{ \circ } \approx 0.84F_{e} } \hfill \\ \end{array} } \right.$$

The interface force *F*_*i*_ is undertaken by the east main sliding Zone IV. Since there is a 5° angle between the force and the vertical line of middle interface, the force can be decomposed into the tangential force *F*_*i*_ and the pressure *F*_*n*_. Simultaneously, when the west slope is destroyed, the thrust *F*_*t*_ causes the dislocation of massess along the interface and produce a friction *F*_*d*_ on Zone IV. *F*_*l*_ and *F*_*n*_ can be expressed as follows:

2 $$\left\{ {\begin{array}{*{20}l} {F_{n} = {\text{F}}_{i} \cos 5^{ \circ } \approx {\text{F}}_{i} } \hfill \\ {F_{l} = {\text{F}}_{i} \sin 5^{ \circ } \approx 0.09{\text{F}}_{i} } \hfill \\ \end{array} } \right.$$

By the orthogonal decomposition of the three forces, the volume force acting on the Zone IV can be expressed as follows:

3 $$\left\{ {\begin{array}{*{20}l} {\sum {X = {\text{F}}_{nx} - {\text{F}}_{lx} - {\text{F}}_{dx} = {\text{F}}_{n} \cos 28^{ \circ } - ({\text{F}}_{l} + {\text{F}}_{d} )\sin 28^{ \circ } \approx 0.83{\text{F}}_{i} - 0.47{\text{F}}_{d} } } \hfill \\ {\sum {{\text{Y}} = {\text{F}}_{ny} - {\text{F}}_{ly} - {\text{F}}_{dy} = {\text{F}}_{n} \sin 28^{ \circ } + ({\text{F}}_{l} + {\text{F}}_{d} )\cos 28^{ \circ } \approx 0.55{\text{F}}_{i} - 0.88{\text{F}}_{d} } } \hfill \\ \end{array} } \right.$$

From Eqs. (1)–(3), it can be seen that the landslide thrust *F*_t_ and its derivative *F*_*e*_, *F*_*d*_ have a large component along the *y*-axis, indicating that the east slope is mainly pushed by *F*_*t*_ along its sliding direction. This force is likely to cause the extrusion failure of the front excavated slope and the imbalance of the force moment. The horizontal component of the interface force *F*_*i*_ is larger than its vertical ones, which mainly acts on the east main sliding area, and cooperates with the landslide thrust to expand the damage range.

### Simulation scheme

Since the landslide volume force acts on the horizontal and vertical directions, it is beneficial to establish a three-dimensional (3D) numerical model and analyze the slope failure process by the finite element method. Meanwhile, although it is difficult to calculate the lateral thrust directly, the numerical technology can be applied to obtain the displacement, stress and strain characteristics, and the strength reduction method can be used to calculate the factor of the slope safety under certain conditions. Furthermore, two simulation schemes are proposed. Firstly, to model the progressive failure of the entire slope area, the whole model of the large-scale slope is established, and the stability of the entire slope area under the combined disturbance of excavation and rainfall is analyzed. Simultaneously, for the whole model, after the initial geo-stress balance, only one construction stage is considered when analyzing the instability affected by excavation and rainfall. Secondly, with the aim of understanding the detailed deformation localization and local failures, the east and west sub-slope models were built up individually. The contact stresses acting on the common nodes of the interface elements are extracted when the west slope is damaged and applied to the east slope when simulating the impact of the lateral thrust.

**Fig. 6:**
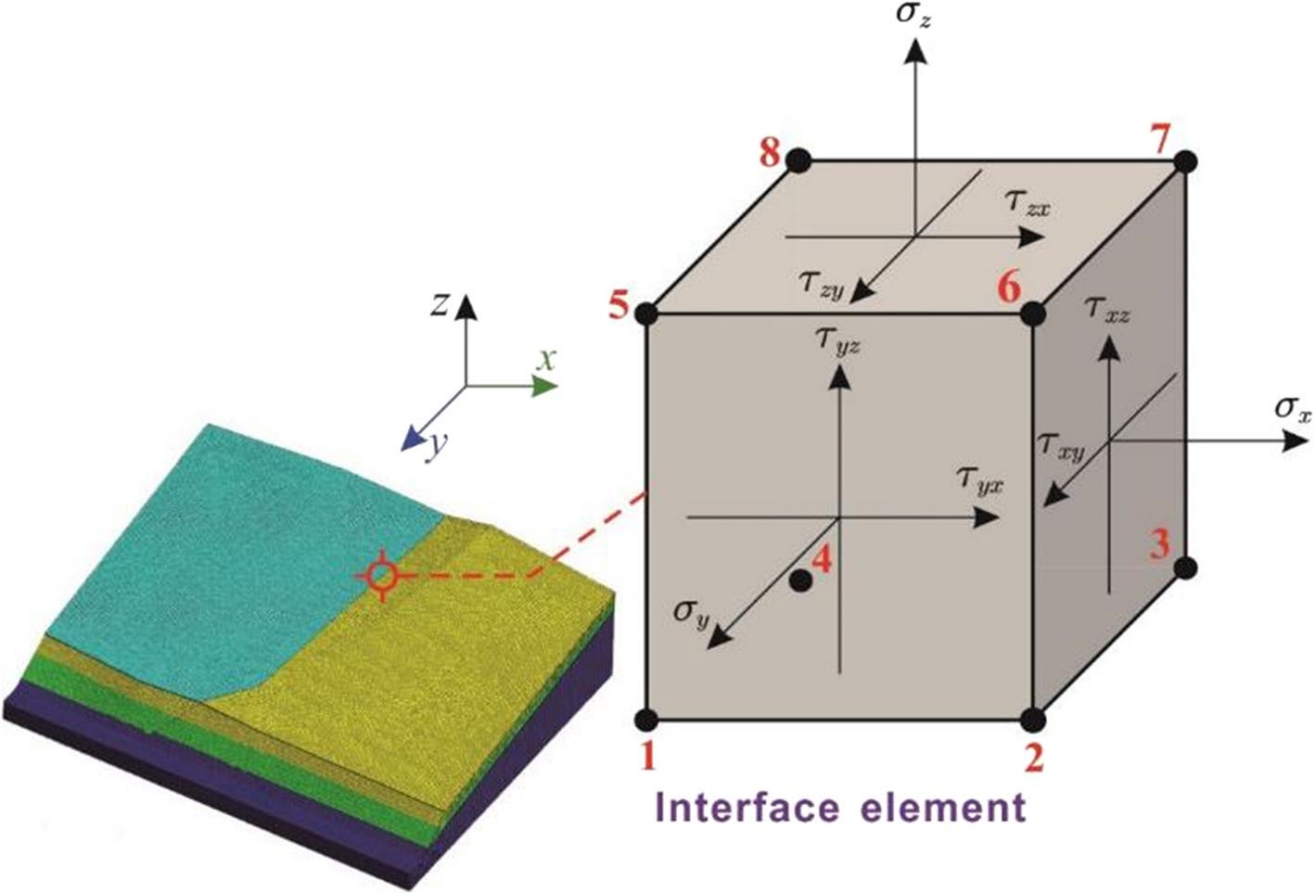
The stress state of an 8-node regular hexahedral element

For the numerical models during the simulation scheme, the lateral thrust is simulated through transmitting the stresses acting on the common nodes of the interface elements between the two sub-slopes. In this study, the contact elements at the interface between the two adjacent areas are the 8-node hexahedral elements. When the west slope losses its stability, the lateral landslide thrust will be generated and transferred through the interface elements. The suffered normal stress and the shear stresses of the interface elements are shown in Fig. 6.

**Fig. 7:**
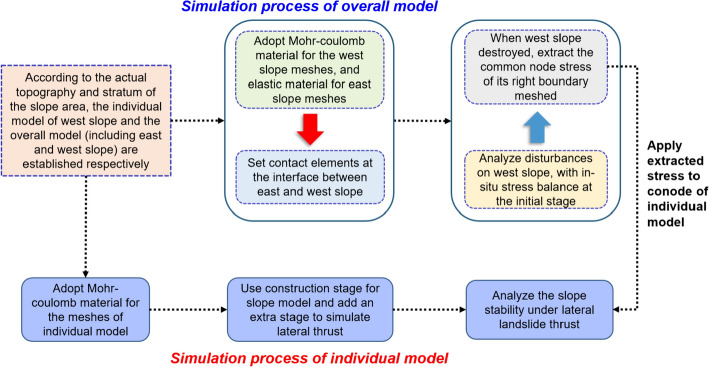
Three-dimensional stability analysis of the large-scale slope under lateral thrust

Namely, through extracting the normal stress and the shear stress acting on the common nodes when the west slope is fully damaged and applying them onto the east slope. The simulation of lateral thrust can be realized. For the used finite element program, the displacements of element nodes are treated as the basic unknowns, and the relationship between element stress and strain can be expressed as:

4 $$\left\{ {\begin{array}{*{20}l} {\sigma_{x} } \hfill \\ {\tau_{xy} } \hfill \\ {\tau_{xz} } \hfill \\ \end{array} } \right\} = \frac{E}{(1 + v)(1 - 2v)}\left[ {\begin{array}{*{20}l} {1 - v} \hfill & 0 \hfill & 0 \hfill \\ 0 \hfill & {\frac{1 - 2v}{2}} \hfill & 0 \hfill \\ 0 \hfill & 0 \hfill & {\frac{1 - 2v}{2}} \hfill \\ \end{array} } \right]\left\{ {\begin{array}{*{20}l} {\varepsilon_{x} } \hfill \\ {\gamma_{xy} } \hfill \\ {\gamma_{xz} } \hfill \\ \end{array} } \right\}$$

where *E* is the elastic modulus; *v* is the Poisson's ratio; $\varepsilon_{x}$, $\gamma_{xy}$ and $\gamma_{xz}$ are the normal strain and shear strain, respectively. The stress is given a first-order relationship with the strain through the elastic modulus and Poisson's ratio, and the correlation between the element strain and node displacement can be expressed as:

5 $$\varepsilon_{x} = \frac{\partial u}{{\partial x}},\quad \gamma_{xy} = \frac{\partial v}{{\partial x}} + \frac{\partial u}{{\partial y}},\quad \gamma_{xz} = \frac{\partial v}{{\partial x}} + \frac{\partial u}{{\partial z}}$$

where *u* and *v* represent the displacements of the nodes along the *x*, *y* and* z* directions. Namely, the element strain and stress can be determined by the first and second derivatives of the displacements of its nodes. To simulate the influence of the west slope failure on the east slope, it is necessary to ensure the simulated sliding deformation of the slope model consistent with the actual situation. According to the field investigation, the rock and soil mass was dislocated, and the pinnate cracks occurred at the interface. When the west slope model is simulated separately, the displacement constraint will be imposed along the interface. To detailed simulation process is shown in Fig. 7, and the specific steps are explained as follows:

1. The individual model of the east slope and the overall model including the west and east sub-slopes are established, respectively. The Mohr–Coulomb criterion is applied to the individual east model and the west sub-slope in the overall model. The elastic material is assigned to the east sub-slope in the overall model, and its mesh and stratum division are the same as the individual east model. However, the elastic modulus of each stratum of the east sub-slope in the overall model is set to be maximum. At the same time, the interface elements are set between the two sub-slopes.
2. During the construction stage, the deformation of the west slope affected by external factors in the overall model will be analyzed. The initial working condition is added after the in-situ stress balance. Since the elastic modulus of the east sub-slope at this state is maximum, it can be treated as a rigid body and will not affect the stability calculation of the west slope. The interface pressure generated by the west slope failure will not change due to the deformation of the rigid east sub-slope.
3. The construction stage is also set for the individual east slope model, and it will be activated at the end of the simulation of the lateral thrust. When the west slope fails, the normal stress and the shear stress of the common nodes of the interface elements will be extracted and import onto the corresponding nodes of the individual model as the boundary condition. Note that this boundary condition is passivated during the first few construction stages and activated in the last stage to simulate the lateral thrust on the east slope.

In this way, the impact of the lateral landslide on east slope can be simulated effectively. Since the rigid east sub-slope does not participate in the strength reduction calculation, the safety factor of the west sub-slope will be calculated using the overall model. Then, the sliding deformation of the east slope is analyzed by the individual model. Furthermore, the lateral landslide thrust, excavation and rainfall are set as different construction stages, the influence of each disturbance factor on the slope can be analyzed effectively. Depending on the obtained results, the failure mechanism of the slope subjected to the varying disturbance factors can be investigated.

### Parameter back analysis

#### Back analysis of slope stability

Considering that the slope is affected by the soil arch effect (Liu et al. 2021a, b), both schemes require the 3D numerical simulation. The range of the factor of slope safety can be determined in advance. After the two slope failures, the geomorphology characteristics, such as the front shear outlets and the rear tension cracks, were fully measured. Although the sliding body formed, but there was no large-scale dumping of soil and rock. Wang et al. (2017) summarized the corresponding relationship between the slope deformation characteristics and the safety factors and suggested that the deformation of the cutting slope can be divided into the creeping stage (1.05 < *F*_*s*_ < 1.1), the extrusion stage (1.02 < *F*_*s*_ < 1.05), the sliding stage (0.98 < *F*_*s*_ < 1.02) and the sudden slip stage (0.95 < *F*_*s*_ < 0.98). According to their study, the safety factor of the east and west sub-slopes is between 0.98 and 1.02. The saturated strength parameters of soils under the water level are determined based on this safety factor range and geological data. Then, they are applied for the subsequent simulation. If the deformation characteristics obtained by the simulation are in good agreement with the actual slope failure, the effectiveness of the stability back analysis will be validated.

#### Inversion of shear strength parameters

The inverse calculation of the shear strength parameters of the slope materials is based on the limit equilibrium method (Nguyen 1984; Ishii et al. 2012; Shinoda et al. 2019). Assuming that the slope is in the critical state, the balanced relationship between the sliding and anti-sliding forces along the corresponding sliding surface will be used to back-calculate the soil shear strength indices. In this study, the slope/W module of the GeoStudio software was applied to perform the parameter inversion based on the limit equilibrium method. To reveal the potential influence factors of the slope failure, two geological sections (K158 + 500 and K158 + 645) were selected for inversion, The Spencer stability analysis method and the sliding surface optimization option were adopted, and the entry and exit of the sliding surface were set according to the on-site failure characteristics.

Meanwhile, the safety factor range (0.98 < *F*_*s*_ < 1.02) was used as the inversion target, and the saturation density and shear strength parameters determined during the geological survey were used as the initial values. The cohesion and internal friction angle of rock and soil mass in the water-level changing area was reduced by a certain proportion. When the calculated position of the critical sliding surface and the safety factor were consistent with the field observation, the calculation was stopped, and the shear strength parameters were recorded. Based on the geological prospecting data, the physical and mechanical parameters of rock and soil mass are shown in Table 1.

**Table 1:** Physical and mechanical parameters of the geo-materials

Formation	Deformation modulus (kPa)	Poisson’s ratio	Weight (kN/m^3^)	Cohesive force *C* (kPa)	Internal friction angle *φ* (**°**)
Plain fill	2.7 × 10^4^	0.28	20	15	15
Sandy mudstone	1.5 × 10^5^	0.31	21	29.4	19
Carbonaceous shale	6.1 × 10^5^	0.27	22 (23.5)	37	24
Argillaceous limestone	8.3 × 10^5^	0.29	23.5 (24)	80	31

**Fig. 8:**
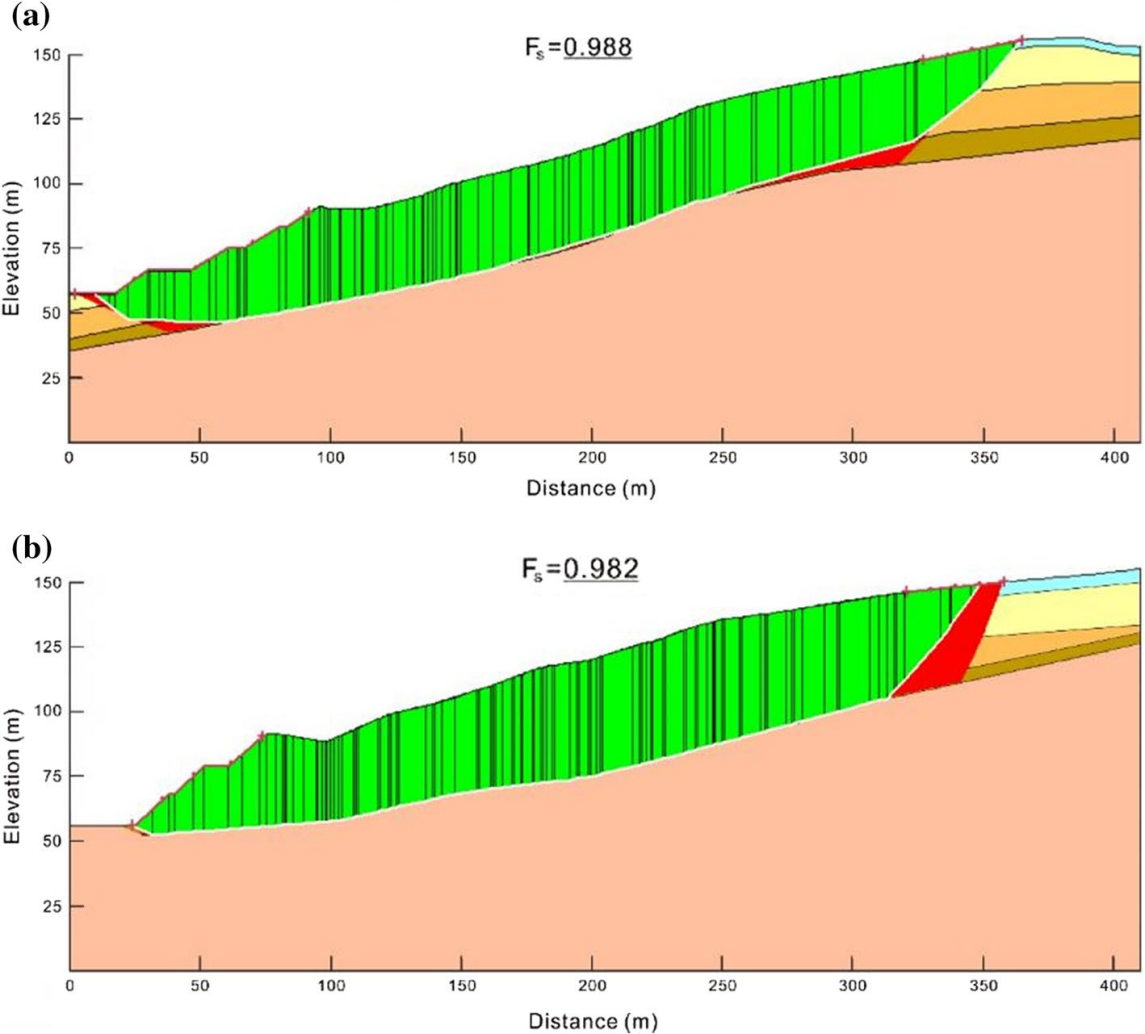
The final sliding surfaces obtained by inversion analysis: **a** the K158 + 500 section and **b** the K158 + 645 section

According to the geological survey, the water-level changing areas of the K158 + 500 and K158 + 645 sections contain carbonaceous shales and argillaceous limestones. Since the argillaceous limestones did not slip, only the parameters of carbonaceous shales were inverted. Referring to the experimental results on the saturated shear strength of carbonaceous shales (Zhou 2003; Liu et al. 2015), both the cohesive force and internal friction angle can be reduced by the water action, and the reduction rate of the former can be five times higher than the latter. Based on this ratio, the shear strength parameters were inversed, and the dichotomy concept was applied to make the calculated safety factor approach the targeted inversion range. The final sliding surfaces obtained by the inversion analysis are shown in Fig. 8, and the inverse saturation parameters are listed in Table 2. When the shear strength parameters are *C* = 25 kPa and *φ* = 20.5°, the critical safety factors obtained from the calculation of the two sections are 0.988 and 0.982, respectively, both of which are located in the targeted inversion range. The most dangerous slip surface obtained from the study is consistent with the field investigation. Therefore, this group of parameters is selected for subsequent simulations.

**Table 2:** Parameter back-calculation of saturated carbonaceous shales

Reduction ratio between *C* and *φ*	*C* (kPa)	*φ* (°)	Safety factor of K158 + 500 section	Safety factor of K158 + 645 section
Initial parameters	37	24	1.214	1.171
5:1	32	23	1.143	1.123
	27	22	1.097	1.064
	25	21	1.055	1.029
	22.5	20.5	1.029	1.009
	20	20	0.988	0.982

### Model establishment and calculation scenarios

#### Establishment of the 3D slope model

According to the slope topographic map, the contour data were extracted and imported into the Surfer software to obtain the three-dimensional slope surface. Then the stratum and water level were determined based on the geological survey, and the excavation area was restored according to the elevation. The Midas finite element software was used to establish the whole model of the actual large-scale slope according to the first numerical simulation scheme. In addition, an individual mode (east slope) and an overall model (including the east and west sub slopes) were established according to the second numerical simulation scheme, in which the former was assigned with Mohr–Coulomb material, and the east slope of the latter was considered to be rigid, while the west slope was still assigned with the Mohr–Coulomb material. The interface elements were set at the border between two regional slopes of the overall model. The elastic modulus of the rigid east sub-slope was set to be maximum, and the coulomb friction model was used for the interface element. The normal and shear stiffness modulus were set as 1 × 10^7^ kPa and 2 × 10^6^ kPa, respectively, and the cohesion and internal friction angle were set as 10 kPa and 5°, respectively.

The overall model and the east slope model both used eight-node hexahedral elements. The former had 162,250 elements, while the latter had 137,748 elements. Fixed bearings were applied to the bottom boundary (*x*, *y*, *z*-direction constraints), while the left and right boundaries were supported by vertical sliding bearing (*x*-direction constraint). There were no constraints in the front and back, nor at the middle interface of the overall model to ensure that the elements can deform.

#### Excavation and rainfall analysis conditions

The rainfall infiltration was simulated by replacing the shear strength parameters of carbonaceous shales in the area of water-level changing with saturation parameters. In the first scheme, the simulation sequence was initial geo stress balance → combined excavation and rainfall, the excavated soil mass and the parameters of the two regional slopes were passivated and replaced at the same time. In the second simulation scheme, the construction order of the individual model was initial geo stress balance → excavation → rainfall → lateral thrust, and the sequence for the overall model was initial geo stress balance → excavation → rainfall. The overall model was firstly analyzed. The normal stress and shear stress of the common elements at the right boundary of the west slope after the rainfall stage were extracted and imported onto the individual model as the load boundary condition and activated during the lateral loading stage to simulate the influence of lateral landslide thrust.

## Results and analyses

### Deformation and stress characteristics of the whole slope

**Fig. 9:**
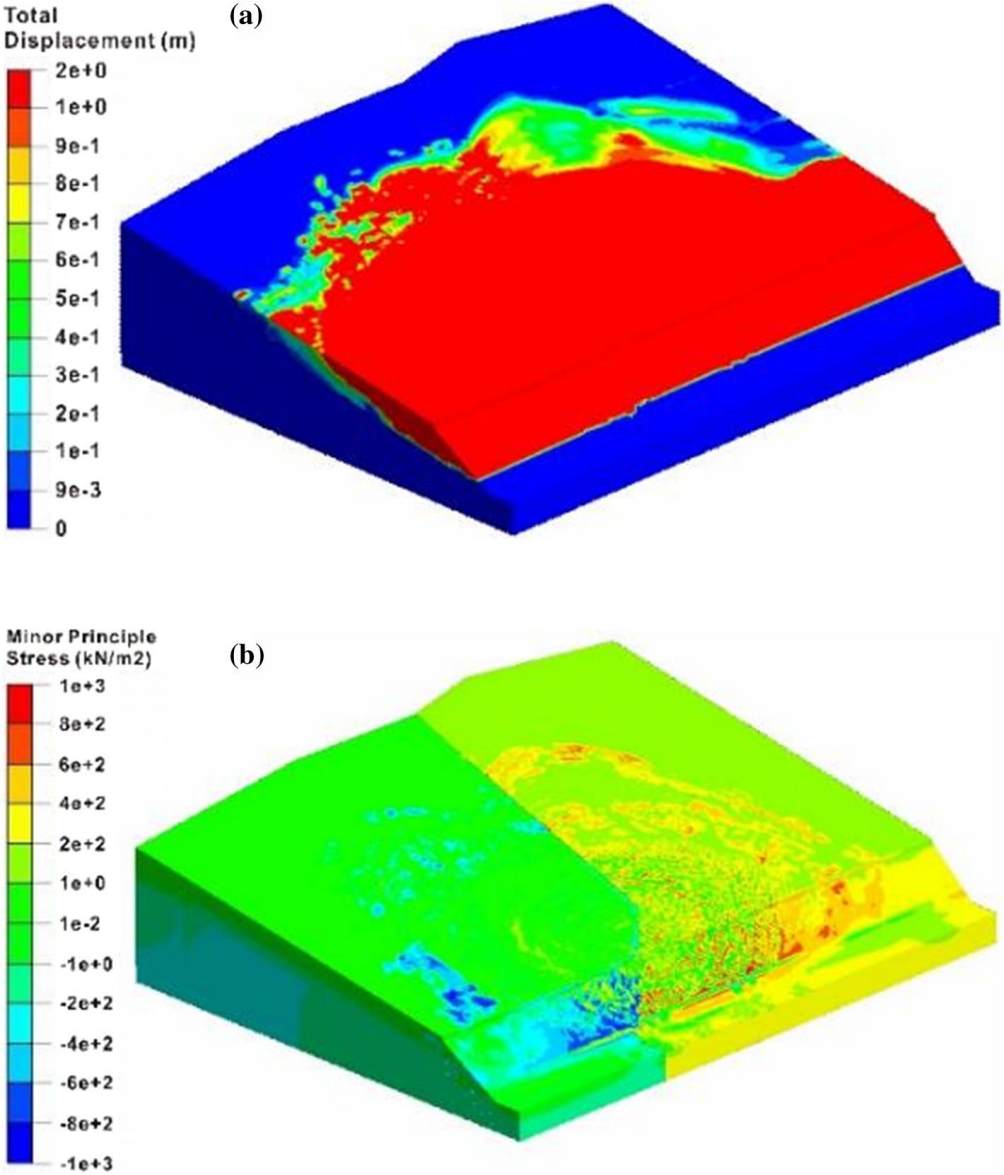
Failure characteristics of the whole slope: **a** total displacement contour and **b** minimum principal stress contour

According to the analysis results of the whole model, the safety factor of the large-scale slope under the combined excavation and rainfall is 0.99, and the overall instability mode occurs, as shown in Fig. 9. Comparing the total displacement distribution with the remote sensing image, the simulated failure area is basically the same as the range of the observed landslide body. The average total displacement of 1.78 m at the rear of the west slope is also close to the 1.8 m at the same location based on the field investigation, indicating that the numerical simulation captures the main deformation features of the slope. Furthermore, the stress characteristics of two regional slopes are obviously different. The minimum principal stress of the west sliding mass demonstrates the local tensile failures, while the minimum principal stress at the east side shows the characteristics of extrusion deformation. Because the overall model cannot be used to calculate the safety factors of the two regional slopes separately, the following will analyze the sliding failure process of the east and west slopes, respectively using the second numerical simulation scheme.

### Failure process and stability of the regional slopes

**Fig. 10:**
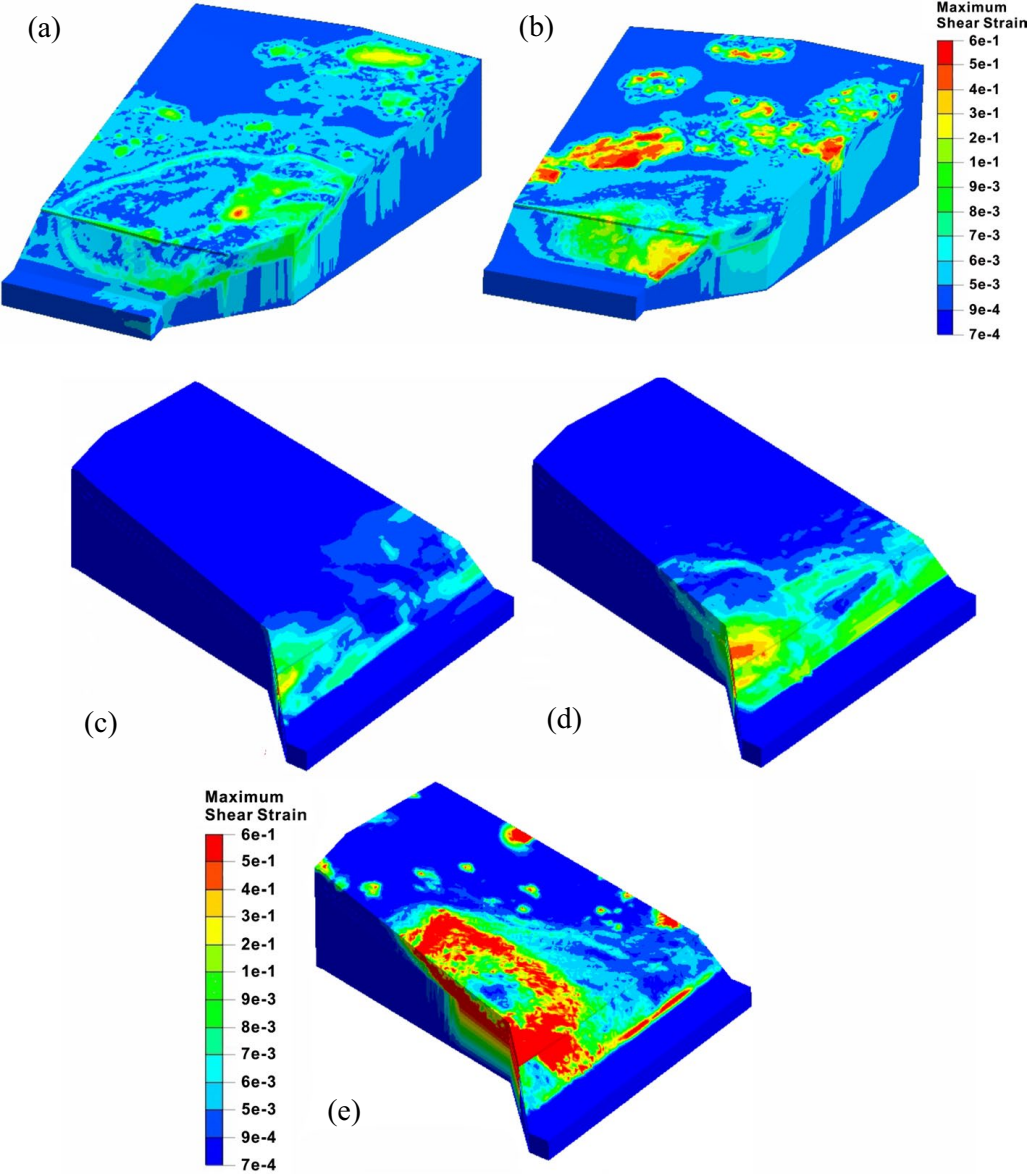
The maximum shear strain contours of the regional slopes under different analysis conditions: the west slope after **a** excavation and **b** rainfall; the east slope after **c** excavation, **d** rainfall and **e** lateral thrust

Figure 10 shows the evolution process of the maximum shear strain filed of the slope. To discuss the influence of the west slope failure on the east slope, the contours of the two regional slopes are separated from the interface. It can be seen from Fig. 10 that the plastic zone along the toe to the middle of the slope firstly forms after the excavation of the west slope. The sliding surface is located at the carbonaceous shale and upper part of argillaceous sandstone. The high stresses are concentrated at the middle, back, and the excavation surface of the slope. The front plastic zone is connected with the rear through the interface of carbonaceous shale and argillaceous limestone, showing the feature of bedding slip.

Before the lateral thrust stage, the sliding deformation only occurs at the excavated area of the east slope. The plastic zone at the slope front is concentrated with a small part of the relaxation deformation zone of the soil masses near the excavation surface. In the rainfall stage, the plastic zone extends rapidly along the interface to the middle, and the high stresses are concentrated from the toe to the left side of the excavated slope. In the lateral thrust stage, the plastic zone penetrates to the rear of the slope, and a large-scale high-stress concentrated zone occurs in the left slope body and the lower part, and finally, a triangular landslide body forms.

**Fig. 11:**
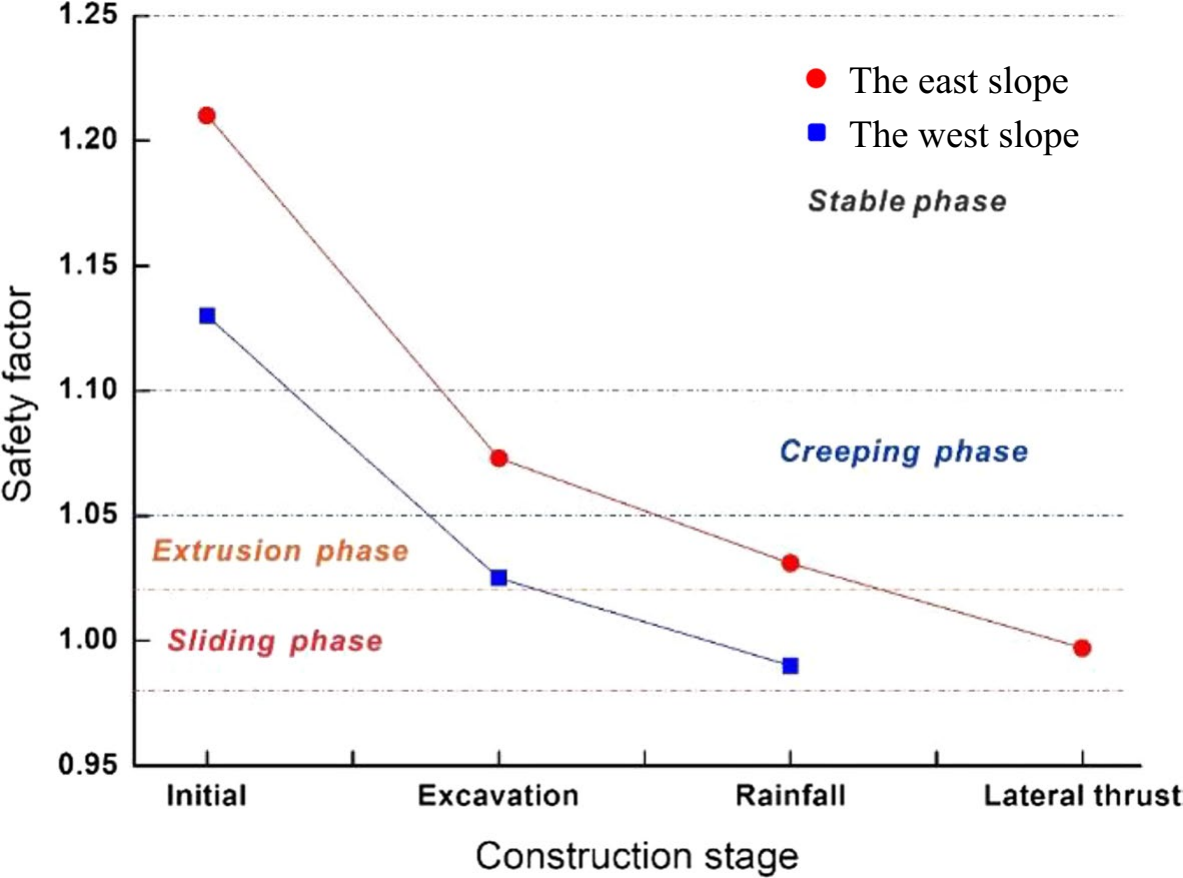
The factor of slope safety under different analysis conditions

Figure 11 shows the variation of the factor of slope safety in each modeling stage. The stability of the east and west slopes gradually decreased with the construction continuing. The safety factor of the west slope in the rainfall stage is 0.99, which is in the targeted range of the safety factor determined by the stability back analysis (0.98 < *F*_*s*_ < 1.02), and also agrees with the parameter inversion results, verifying the satisfactory accuracy of the numerical simulation and inversion analysis. Based on the four deformation stages summarized by Wang et al. (2017), the stability evolution of the west and east slopes can be concluded in Fig. 11.

### Failure characteristics of the west slope

**Fig. 12:**
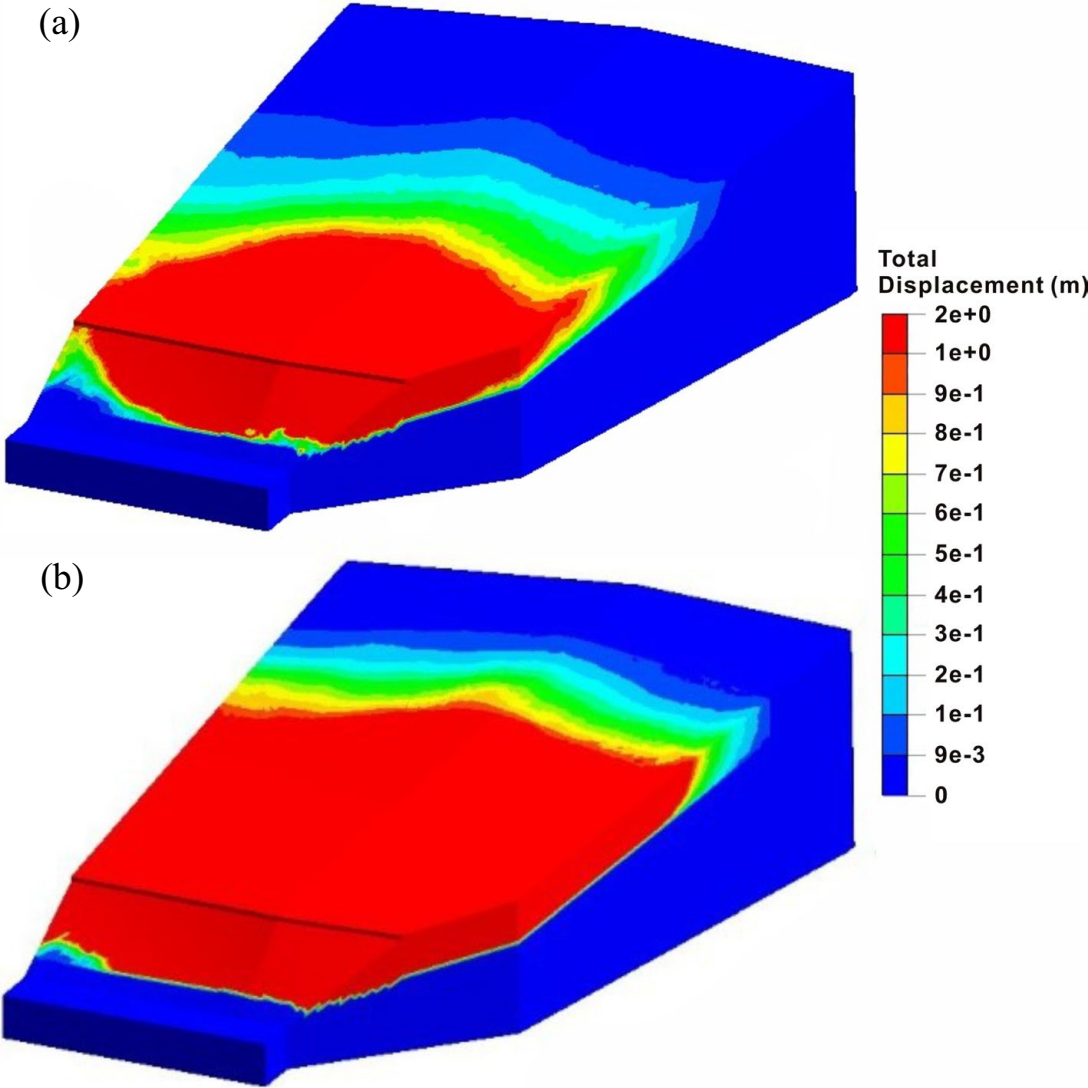
Total displacement characteristics of the west slope: after **a** excavation and **b** rainfall

Affected by the upper thick and soft soil strata, the deformation of the west slope during the construction period is relatively intense. Before excavation, the safety factor of the west slope is 1.13, which is in a stable state. After excavation, its safety factor drops sharply to 1.025. The west slope directly enters the extrusion stage, forming a landslide body that connects from the toe to the middle of the slope, as shown in Fig. 12. The excavation makes the strata fully exposed at the leading edge of the slope, which promotes the formation of landslide shear outlet, and also makes the slope lose the critical anti-slide part. In the rainfall stage, the safety factor reaches 0.99, and the slope enters the sliding stage. The rise of groundwater level changes the original linear sliding into the bedding landslide along the interface between carbonaceous shale and argillaceous limestone, and the failure area expands backward. The stress concentration phenomenon occurs at the rear of the slope, showing a typical tensile-shear failure. The simulated rear edge of the sliding body is consistent with the observed tension cracks on site, and the maximum displacement of 1.93 m in Fig. 12b basically agrees with the measured maximum value of 2 m at the trailing edges.

**Fig. 13:**
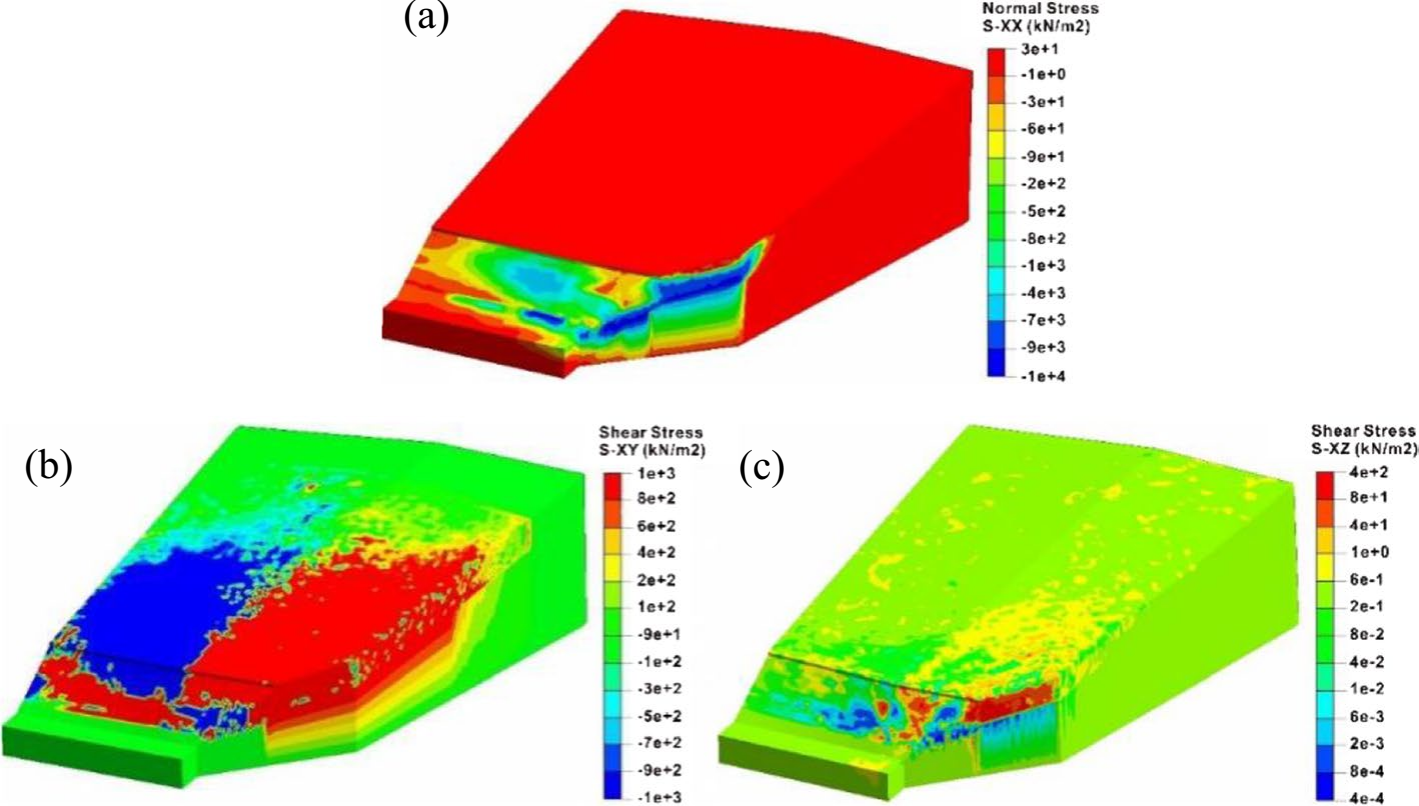
The stress contours of the west slope after rainfall: **a** normal stress along the *x* axial, **b** shear stress in the *xy* plane and **c** shear stress in the *xz* plane

Figure 13 shows the normal principal stress along the *x* axis and the shear stress in the *xy* and *xy* planes in rainfall stage. Regarding the coordinate system, the south represents the positive direction of the *y*-axis, the east represents the positive direction of the *x*-axis, and the vertical direction of the *xy* plane is the *z* axis. After the slope gets damaged, the extreme values of the normal stress and shear stress are located in the front of the junction. The high normal stresses in this area mainly occur at the toe of the west slope, indicating that the landslide thrust generated by the west slope failure mainly squeezes the toe of the east slope. In the middle and rear part of the slope, there is no obvious extrusion deformation perpendicular to the interface. However, the large deformation occurs at the toe of the east slope along the *y* axis because of the force component of the thrust *F*_*t*_ along the sliding direction, demonstrating that the thrust *F*_*t*_ greatly promote the failure evolution of the east slope.

### Failure characteristics of the east slope

**Fig. 14:**
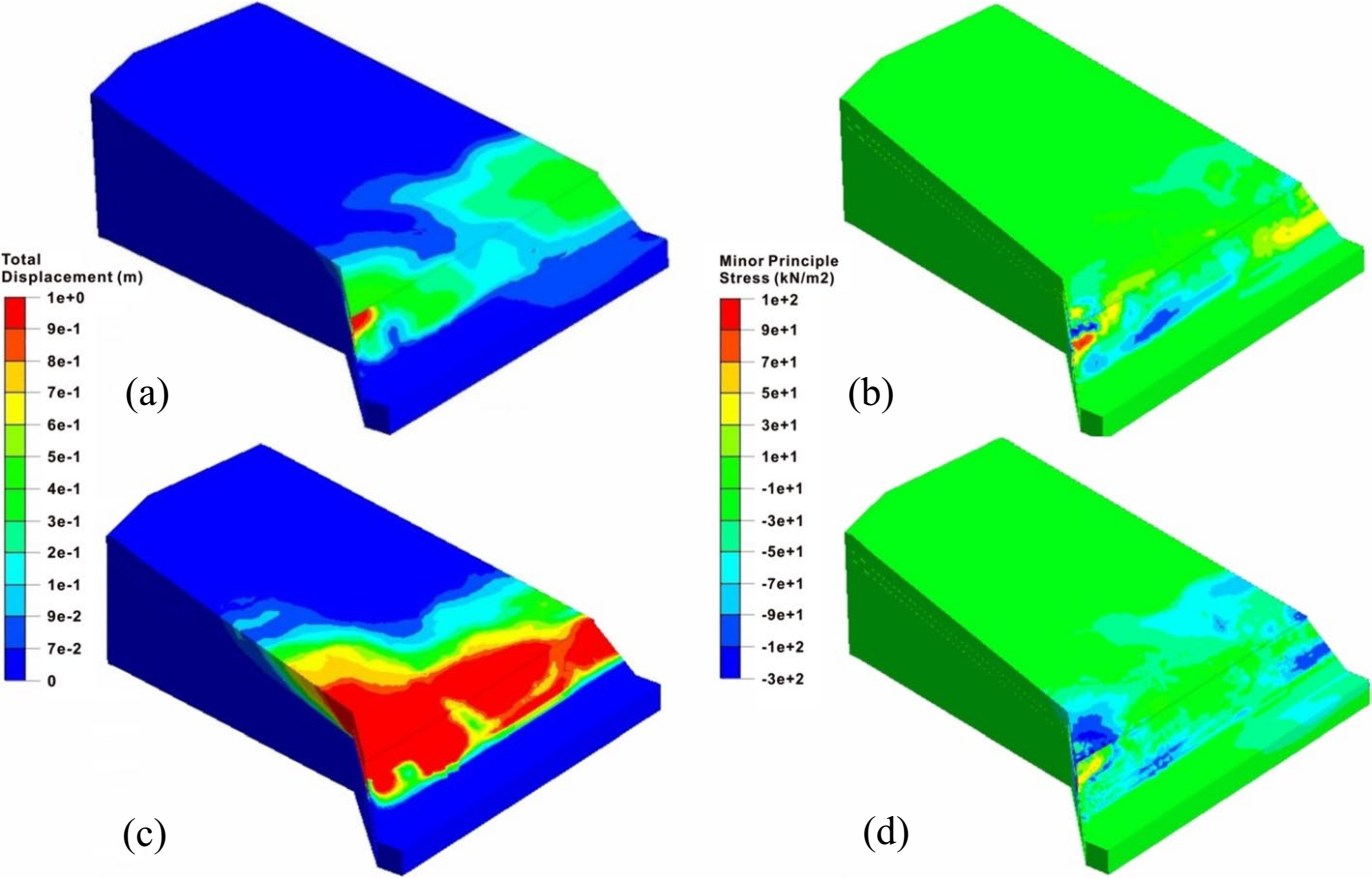
The deformation and stress characteristics of the east slope: total displacement contours after **a** excavation and **c** rainfall; minor principle stress contours after **b** excavation and **d** rainfall

Compared with the severe sliding deformation of the west slope, the east slope shows completely different failure modes before and after the lateral thrust stage. Before excavation, the slope is stable with a safety factor of 1.21. After excavation, the slope enters the creep deformation stage with the safety factor decreasing to 1.073. The unloading effect causes the large local deformation at the front of the slope, and the high tensile stress concentrations phenomena also appears at the slope toe. Besides, the plastic zone is completely connected along the shallow excavated slope. The precursors of the tensile-shear failure of the east slope tend to occur. After the rainfall, the slope enters the extrusion deformation stage, and the safety factor drops to 1.031. The rise of the groundwater level further drives the sliding surface to expand downward. Simultaneously, the softening of the lower carbonaceous shale causes the complete loose of the stable foundation of the excavated slope. Additionally, the obvious shear yield occurs at the toe of the slope due to the high shear stress concentrations, and the whole excavation slope is therefore dragged, and the related tensile-shear failure tends to happen. At this time, the slope shows the characteristics of traction landslide which is similar to the west slope, as shown in Fig. 14.

**Fig. 15:**
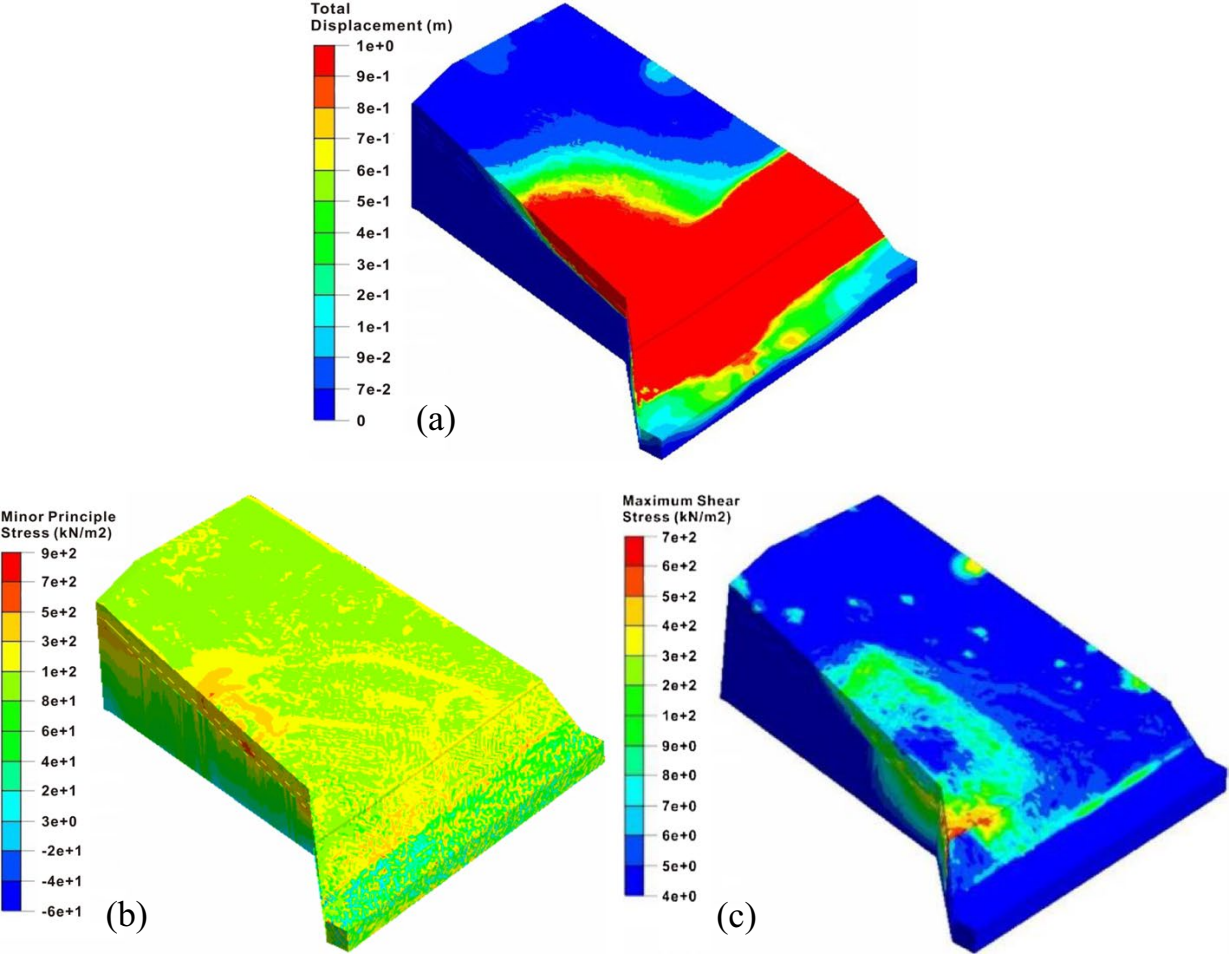
Failure characteristics of the east slope under lateral thrust: **a** total displacement contour, **b** minor principle stress contour and **c** maximum shear stress contour

In the lateral thrust stage, the safety factor of the east slope decreases to 0.997, and it slides as a whole. Under the influence of the volume force of the west slope after its landslide, the east slope failure characteristics in this stage change significantly compared with the previous situation, as shown in Fig. 15a. Firstly, it can be seen from Fig. 15b that most of the minimum principal stresses at the sliding area are positive, and the obvious shear stress concentrations at the rock and soil near the interface are also generated by the failure of the west slope, indicating that the landslide force pushes the front of the slope body forward and results in the typical characteristics of the thrust landslide. This failure mode is completely different from the tensile-shear failure of the excavated slope during the excavation and rainfall stages. Secondly, according to the maximum shear strain contour as shown in Fig. 15c, the lateral thrust induces the local failures at the middle and rear parts of the slope, and the sliding surface is penetrated from the back to the front surface, which shows the forward-sliding mode. Besides, it is quite different from the backward progressive failure mode of the first two stages. Since the west slope cannot produce a large component force along *x* direction, the lateral thrust basically leads to the sliding of the soil near the interface. Meanwhile, although the landslide thrust directly acts on the toe of the east slope and causes the high shear stress concentrations, the excavated slope has been essentially destroyed during the rainfall stage. However, the thrust changes the stress state of the front mass and promote the formation and evolution of the damage range and landslide.

Compared with the contours and landslide characteristics, the simulated damage area of the east slope is basically consistent with the site. The simulated total displacement at slope top is nearly 1 m, which is also consistent with the steep scarp with a maximum height of nearly 1.1 m observed on site. Besides, the maximum displacement of 36 cm in the road area agrees with the phenomenon of road uplift of 40 cm, indicating that the numerical simulation effectively restores the slope failure process.

### Failure mechanism of the slope under lateral thrust

By comparing the numerical and observed deformation and failure characteristics of the two regional slopes, it can be found that the progressive failure process of the regional slopes has been revealed effectively by the developed approach of 'separate simulation and superposition analysis'. Furthermore, the failure mechanism of the east slope under the lateral thrust has been successfully captured. According to the characteristics of deformation localization and sliding surface, it can be proven that the lateral thrust induces the slope to enter the sliding stage by promoting the local large deformation and damage of the excavated slope. Namely, it is the main cause of the final failure of the east slope. Besides, the special boot-shaped appearance of the east slope makes the toe bear the radial and tangential components of the volume force of sliding masses, which significantly enhances the impact of the lateral thrust on the east slope. Before being subjected to lateral thrust, the front part of the east slope deforms due to the engineering excavation disturbance, and the tensile-shear failure tends to occur due to the growth of soil and rock weight and the strength weakening of geo-materials after rainfall, showing the traction characteristics. In the lateral thrust stage, the tangential component force acting on the interface forms a plastic zone in the middle and rear parts, causes the compression-shear failure of the rock and soil mass in this area, and drives the front block to form a pushing mode of landslide. The radial component force acting on the toe leads to the stress redistribution at the front part of the excavated slope and the formation of a landslide hill. During the whole construction period, the sliding surface firstly generates at the front and then develops upwards, showing a backward failure mode. Later, the cracks initiate at the slope back and propagate from back to front, showing a forward failure mode. The front and rear sliding zones connect in the middle, resulting in the composite sliding deformation and failure pattern containing the coupling characteristics of both traction and push.

## Conclusions

To reveal the influence of the lateral thrust generated by the local slope failure on its adjacent slope, a highway construction involving two regional slopes in the east and west in Guangdong Province, China, is taken as an example. The field investigation, theoretical analysis and numerical simulation are conducted to investigate the failure and instability process of the east slope under the lateral thrust. The main conclusions can be obtained as follows:

1. Based on discretization, the lateral thrust generated by regional slope failure can be transformed into the normal and tangential stresses on the shared nodes of the mesh elements at the junction between the adjacent slopes. The slope stability analysis under lateral thrust can be achieved by simulating regional slope and its adjacent slopes separately, and then applying stress to the boundaries. This approach allows for the various disturbance factors on the adjacent slopes to be analyzed appropriately. Furthermore, this method avoids the problem of being unable to determine the safety factor of each regional slope in the overall analysis.
2. The lateral thrust generated by regional slope instability is the combined force of the landslide thrust along the landslide direction and the interface force perpendicular to the direction of the landslide thrust. For the adjacent slope, lateral thrust points towards its interior. The landslide thrust can cause dislocation of adjacent rock and soil and generate the frictional force along the boundary interfaces, breaking the torque balance of the adjacent slope and causing the overall landslide. The interface force directly acts on the adjacent slope, and cooperates with the landslide thrust to expand the damage range. When the front of the adjacent slope blocks regional slope, the landslide thrust will squeeze the front of adjacent slope, causing the uplift of rock and soil masses.
3. In large slopes under excavation and rainfall, the traction landslide failure of a single regional slope can transform the local deformation of its adjacent slope into overall sliding. The squeeze and friction of the adjacent slopes caused by landslides can result in the compression and shear failure of rock and soil, leading to the initiation of plastic zones in the middle and rear of the slope and driving them to expand forward. Eventually, they connect with the traction sliding surfaces caused by the excavated slope due to tension and shear failure, forming a composite landslide with dual characteristics of traction and push types.

## Data Availability

The data underpinning this publication can be accessed from Brunel University London's data repository, Brunelfigshare here under a CCBY licence: 10.17633/rd.brunel.25360690.
